# Diagnostic outcomes of robotic-assisted bronchoscopy for pulmonary lesions in a real-world multicenter community setting

**DOI:** 10.1186/s12890-023-02465-w

**Published:** 2023-05-09

**Authors:** Faisal Khan, Joseph Seaman, Tina D. Hunter, Diogo Ribeiro, Balaji Laxmanan, Iftekhar Kalsekar, Gustavo Cumbo-Nacheli

**Affiliations:** 1 Franciscan Health Indianapolis, Indianapolis, IN USA; 2 Sarasota Memorial Health Care System, Sarasota, FL USA; 3 CTI Clinical Trial and Consulting Services, Covington, KY 41011 USA; 4 Lung Cancer Initiative, Johnson & Johnson, New Brunswick, NJ USA; 5 Spectrum Health, Grand Rapids, MI USA

**Keywords:** Biopsy, Lung cancer, Lung lesion, Robotic bronchoscopy

## Abstract

**Background:**

Robot-assisted bronchoscopy (RAB) is among the newest bronchoscopic technologies, allowing improved visualization and access for small and hard-to-reach nodules. RAB studies have primarily been conducted at academic centers, limiting the generalizability of results to the broader real-world setting, while variability in diagnostic yield definitions has impaired the validity of cross-study comparisons. The objective of this study was to determine the diagnostic yield and sensitivity for malignancy of RAB in patients with pulmonary lesions in a community setting and explore the impact of different definitions on diagnostic yield estimates.

**Methods:**

Data were collected retrospectively from medical records of patients ≥ 21 years who underwent bronchoscopy with the Monarch® Platform (Auris Health, Inc., Redwood City, CA) for biopsy of pulmonary lesions at three US community hospitals between January 2019 and March 2020. Diagnostic yield was calculated at the index RAB and using 12-month follow-up data. At index, all malignant and benign (specific and non-specific) diagnoses were considered diagnostic. After 12 months, benign non-specific cases were considered diagnostic only when follow-up data corroborated the benign result. An alternative definition at index classified benign non-specific results as non-diagnostic, while an alternative 12-month definition categorized index non-diagnostic cases as diagnostic if no malignancy was diagnosed during follow-up.

**Results:**

The study included 264 patients. Median lesion size was 19.3 mm, 58.9% were peripherally located, and 30.1% had a bronchus sign. Samples were obtained via Monarch in 99.6% of patients. Pathology led to a malignant diagnosis in 115 patients (43.6%), a benign diagnosis in 110 (41.7%), and 39 (14.8%) non-diagnostic cases. Index diagnostic yield was 85.2% (95% CI: [80.9%, 89.5%]) and the 12-month diagnostic yield was 79.4% (95% CI: [74.4%, 84.3%]). Alternative definitions resulted in diagnostic yield estimates of 58.7% (95% CI: [52.8%, 64.7%]) at index and 89.0% (95% CI: [85.1%, 92.8%]) at 12 months. Sensitivity for malignancy was 79.3% (95% CI: [72.7%, 85.9%]) and cancer prevalence was 58.0% after 12 months.

**Conclusions:**

RAB demonstrated a high diagnostic yield in the largest study to date, despite representing a real-world community population with a relatively low prevalence of cancer. Alternative definitions had a considerable impact on diagnostic yield estimates.

**Supplementary Information:**

The online version contains supplementary material available at 10.1186/s12890-023-02465-w.

## Introduction

The field of guided bronchoscopy is continually evolving, driven by the need to efficiently and safely sample pulmonary lesions and improve diagnostic accuracy [1, 2]. Technological advancements have included radial endobronchial ultrasound (R-EBUS) imaging, electromagnetic navigation (EMN), virtual, and ultrathin bronchoscopy [1, 2], as well as newer radiographic imaging technologies. 

The use of guided bronchoscopy technologies to diagnose pulmonary lesions has significantly improved the diagnostic yield when compared to traditional bronchoscopic biopsy [3]. Nonetheless, the diagnostic accuracy of guided bronchoscopy tools remains suboptimal, particularly for smaller lesions ≤20 mm [3, 4]. The number of smaller peripheral lesions requiring biopsy is expected to increase in the coming years as lung cancer screening programs become more widespread [5]. Being able to successfully biopsy these lesions and correctly establish a diagnosis may thus prove critical to identifying early-stage lung cancer and improving health outcomes. 

Robot-assisted bronchoscopy (RAB) is among the most recent advancements in guided bronchoscopy technology. The Monarch^®^ Platform (Auris Health, Inc., Redwood City, CA), designed to provide bronchoscopic visualization of and access to the patients’ peripheral airways for diagnostic and therapeutic procedures, has received United States (US) Food and Drug Administration clearance and is commercially available in the US [6]. 

Previous studies have demonstrated the feasibility and safety of RAB performed with the Monarch Platform in patients undergoing diagnostic bronchoscopy for indeterminate pulmonary nodules [7–10]. Estimates of diagnostic yield have compared favorably to those of existing devices for guided bronchoscopy, ranging from 74% to 77% after 12 months of follow-up [9, 10]. Despite encouraging results, prior studies have primarily been conducted at academic centers, potentially limiting the applicability of the available data on diagnostic performance and safety in the broader real-world setting. In addition, there is currently no standardized definition for diagnostic yield of bronchoscopy. Studies often differ in the categorization of non-malignant results, consideration of follow-up data, and handling of missing or insufficient diagnostic data. In recent analyses, variability in diagnostic yield calculation methodologies has been shown to cause large differences in computed estimates [11, 12]. Coupled with heterogeneity in study populations, lesion characteristics, and study settings, this variability impairs our ability to perform valid comparisons across studies and as a result, to accurately identify bronchoscopic technologies that improve diagnostic capabilities.

The objective of our study was to evaluate diagnostic yield of the Monarch RAB platform in a multicenter community setting. We also sought to explore the impact of varying diagnostic yield definitions and the factors associated with diagnostic yield and complications of RAB.

## Study design and methods

This chart-review study was conducted at three community hospitals in the US. Patients aged 21 years or older who underwent RAB with Monarch for the biopsy of pulmonary lesions between January 1^st^, 2019 and March 31^st^, 2020 during their routine clinical care were retrospectively identified for inclusion based on an a priori study protocol. 

The medical records of each patient were reviewed to collect data on baseline patient, lesion, and procedure characteristics using structured data collection forms. Characteristics were collected for the primary pulmonary lesion and one secondary lesion, as applicable, per patient. The index diagnosis – based on the pathology results of the RAB-obtained biopsy samples – and any subsequent diagnostic evaluations performed within 12 months in patients without an initial malignant diagnosis were captured. Changes in lesion size from baseline, death, and device- or procedure-related complications occurring during or up to 12 months post-RAB were also recorded.

Bronchoscopy results at index were categorized according to a standardized guide (Additional File Table 1). Per this guide, diagnoses were classified as malignant or non-malignant, with non-malignant results further categorized into three groups based on commonly reported clinical approaches. The first group included those with a specific benign diagnosis (e.g., infection, granuloma). The second group included patients with a non-specific benign finding (e.g., inflammation) on biopsy. The third group included patients with a biopsy result that was unlikely to explain the presence of a pulmonary nodule (e.g., normal lung parenchyma, bronchial cells) who were therefore considered non-diagnostic. 

The bronchoscopy procedures were performed by six operators, of whom four had no prior experience with the RAB platform. Operators included specialists of both interventional pulmonology and thoracic surgery.

All methods were performed in accordance with the ethical guidelines of the 1975 Declaration of Helsinki. The protocol was approved, and a waiver of informed consent was granted by the WCG Institutional Review Board (20212331). The study was determined to be exempt from review and a waiver of informed consent was granted Spectrum Health Institutional Review Board (2021-051)

### Diagnostic yield 

Diagnostic yield was calculated as the rate of true positives (TPs) and true negatives (TNs) for malignancy. The diagnostic yield of RAB for pulmonary lesions was evaluated at the time of the index procedure and based on data through 12 months. Primary estimates of diagnostic yield were calculated for each timepoint by employing methods that have been reported in key published bronchoscopy studies [8–10, 13–15].

Diagnostic yield at index was calculated using only pathology results available at the time of the index RAB procedure. For the first index method, malignant results were considered TP while both benign specific and benign non-specific samples were considered TN. For 12-month diagnostic yield, all patients with a malignant or a benign specific diagnosis based on the pathology results of the samples collected via the index RAB were categorized as TP or TN, respectively. Pathology results that were non-diagnostic at index were deemed false negatives (FN) for the first 12-month method. In patients with a benign non-specific diagnosis, follow-up data were reviewed to determine whether the patient’s 12-month status corroborated the benign result. If follow-up included a new malignant diagnosis, death due to lung cancer, or malignant pathology results from a repeat biopsy, they were considered FN. Patients were classified as TN if there was a subsequent evaluation without a malignant diagnosis and no lesion progression on radiographic follow-up. Cases without subsequent follow-up or with an increase in lesion size but without a malignant diagnosis were considered inconclusive. 

Inconclusive cases were excluded from the diagnostic yield calculations for a base-case scenario. A high and low estimate were then calculated by assuming they were all TP (best-case) or FN (worst-case).

Additional estimates were calculated to explore the effect of diagnostic yield definition on the reported outcome and to allow valid comparisons with literature that have used these alternative approaches [16, 17].

For the second method of calculating diagnostic yield at index, only patients with a benign specific diagnosis were considered TN, whereas those with benign non-specific diagnoses were deemed non-diagnostic (similar to the AQUiRE study) [16]. In order for this method to accurately replicate the approach used in the AQUiRE study, cases of organizing pneumonia and interstitial lung disease, classified as benign non-specific per our standardized guide (Additional File Table 1), were reclassified as benign specific only for this definition. 

For the second method of calculating 12-month diagnostic yield, follow-up data were also used to classify both benign non-specific and non-diagnostic cases as TN, FN, or inconclusive (similar to the NAVIGATE study) [17].

Best- and worst-case scenarios for these alternative methods were calculated as described for the primary estimates of diagnostic yield. 

### Sensitivity for malignancy 

Sensitivity for malignancy was calculated as TP/(TP+FN) at 12 months. Unlike the 12-month diagnostic yield calculations, all patients without an index malignant diagnosis were followed through 12 months for malignancy. If follow-up revealed a malignancy, patients were categorized as FN. Otherwise, they were considered TN or inconclusive as previously described. A worst-case scenario assumed that all inconclusive cases were FN. 

### Safety 

The safety outcome was any procedure- or device-related complication, including pneumothorax, bleeding event requiring intervention, or other serious complication occurring within 12 months post-index RAB. 

### Statistical analysis

The sizes of primary and secondary lesions were calculated as the mean of the long and short axes dimensions when both were reported and as the long axis dimension otherwise. The patients’ pre-procedure probability of malignancy was estimated using an adapted Mayo Clinic model [18]. Though this model was created for patients without a cancer diagnosis within the previous five years and without a history of lung cancer, we estimated the probability of malignancy for all patients who had non-missing information by capturing prior history of lung cancer in the same risk category as extrathoracic malignancy.

Index and 12-month diagnostic yield, using the first set of definitions, and pneumothorax rates were compared between subgroups of interest using Chi-square or Fisher’s exact tests, respectively. Subgroups were defined based on patient, lesion, and procedural characteristics. Statistical analysis was performed using SAS® software, version 9.4 (SAS Institute Inc., Cary, NC, USA).

## Results

### Baseline patient characteristics

A total of 264 patients who underwent RAB for the biopsy of pulmonary lesions were included in the study. Baseline characteristics of these patients are summarized in Table 1. Mean patient age at the time of the index RAB was 69.5 ± 10.5 years and 56.8% were female. Approximately 11% of patients had prior history of lung cancer and 52% had chronic obstructive pulmonary disease. Most patients (62.5%) had a single lesion visualized on pre-procedure computed tomography (CT) scans (range: 1 to 22 lesions). Approximately half (48.2%) of the patients had a pre-procedure probability of malignancy ≥65%, as estimated using the adapted Mayo Clinic model.

**Table 1:** Baseline patient and primary lesion characteristics

**Patient characteristic**	**Total**
	**(*N* = 264)**
Age at procedure, years	69.5 ± 10.5
Sex, female	150 (56.8%)
White	252 (95.5%)
Body mass index, kg/m^2^ (*N* = 263)	27.4 ± 6.9
Comorbidities/medical history	
Emphysema	114 (43.2%)
Chronic obstructive pulmonary disease	136 (51.5%)
Prior invasive lung procedure or surgery	29 (11.0%)
History of lung cancer	29 (11.0%)
Primary lung cancer	25 (86.2%)
Metastatic lung cancer – solid tumor	4 (13.8%)
History of extrathoracic malignancies	86 (32.6%)
Family history of lung cancer	36 (13.6%)
Family history of extrathoracic cancer	123 (46.6%)
Smoking status (*N* = 262)	
Prior smoker	125 (47.7%)
Current smoker	72 (27.5%)
Never smoked	65 (24.8%)
Number of pulmonary lesions visualized on pre-procedure CT scans	1 (1–22)
Pre-procedure probability of malignancy^a^ (*N* = 253)	60.2 ± 28.1%
Pre-procedure probability of malignancy^a^ ≥ 65% (*N* = 253)	122 (48.2%)
**Primary lesion characteristic**	**Total**
	**(*N* = 264)**
Lesion size^b^, mm	19.3 (3.2–72.5)
Lesion size^b^ < 20 mm	137/264 (51.9%)
Location	
Proximal third	27/263 (10.3%)
Middle	81/263 (30.8%)
Peripheral (outer third of the lung)	155/263 (58.9%)
Distance from closest edge to pleura^c^, mm	12.0 (0.0–90.0)
Lesion lobe location	
Right upper lobe	82/264 (31.1%)
Right middle lobe	17/264 (6.4%)
Right lower lobe	52/264 (19.7%)
Left upper lobe	79/264 (29.9%)
Left lower lobe	34/264 (12.9%)
Visible bronchus leading to lung lesion	78/259 (30.1%)
Nodule type	
Solid	205/264 (77.7%)
Subsolid	59/264 (22.4%)
Pure ground glass (non-solid)	13 (22.0%)
Semi-solid	46 (78.0%)
Margin specifications	
Spiculated	112/253 (44.3%)
Smooth	92/253 (36.4%)
Lobulated	49/253 (19.4%)
Calcification	11/264 (4.2%)

Results are displayed as n (%), mean ± standard deviation, or median (minimum–maximum)

*CT* Computed tomography

^a^Calculated using an adapted Mayo Clinic model

^b^Size was calculated as the mean of the long and short axes dimensions when both were reported and as the long axis dimension otherwise

^c^Based on 210 non-missing values

### Lesion characteristics

Biopsy was attempted for 264 primary and 48 secondary lesions. The median primary lesion size was 19.3 mm (Table 1), with most lesions being peripherally located (outer third of the lung) (58.9%). Approximately 22% (59/264) of primary lesions were subsolid, of which 46 (78%) were semi-solid and 13 (22%) were pure ground glass. A bronchus sign was observed on the pre-procedure CT scan in 30.1% of primary lesions. Secondary lesion characteristics are provided in Additional file Table 2. 

### Procedure characteristics

Most procedures (93.6%) were carried out in an outpatient setting (Table 2). All procedures were performed under general anesthesia. Most patients (83.3%) had a single lesion biopsied during the RAB procedure (range: 0 to 4), with only three having more than two lesions biopsied. The average procedure time (from scope insertion to withdrawal) was 62.3 ± 27.2 minutes. Concurrent imaging was extremely common, particularly fluoroscopy (99.6%) and R-EBUS (93.9%). Nine procedures (3.4%) used cone-beam CT imaging. R-EBUS allowed for the successful localization of the primary lesion in 243 out of the 248 procedures (98.0%) in which this imaging modality was used. The proportions of concentric and eccentric R-EBUS imaging views were similar across the 165 lesions for which image characterization was available (47.9% and 52.1%, respectively). The most commonly used biopsy tools were aspiration needle (96.6%) and biopsy forceps (70.8%).

**Table 2:** Procedural characteristics

Procedural Detail	Total
	**(*N* = 264)**
Outpatient setting	247 (93.6%)
Operator specialty	
Interventional pulmonologist	226 (85.6%)
Thoracic surgeon	38 (14.4%)
General anesthesia	264 (100%)
Monarch Platform software version ≥ 2.1.5	156 (59.1%)
Number of lesions biopsied	1 (0–4)
Procedure duration, minutes	62.3 ± 27.2^a^
Concurrent imaging	
Fluoroscopy	263 (99.6%)
Cone-beam CT	9 (3.4%)
Radial endobronchial ultrasound	248 (93.9%)
Successful localization of primary lesion	243/248 (98.0%)
Concentric view (of cases reporting image characterization)	79/165 (47.9%)
Eccentric view (of cases reporting image characterization)	86/165 (52.1%)
Biopsy tools	
Aspiration needle	255 (96.6%)
Biopsy forceps	187 (70.8%)
Cytology brush	45 (17.1%)
Biopsy samples evaluated by Rapid Onsite Evaluation	172 (65.2%)
Lymph node biopsy	120 (45.5%)

Results are displayed as n (%), mean ± standard deviation, or median (minimum–maximum)

*CT* Computed tomography

^a^Based on 256 non-missing values

### Index diagnosis

**Fig. 1:**
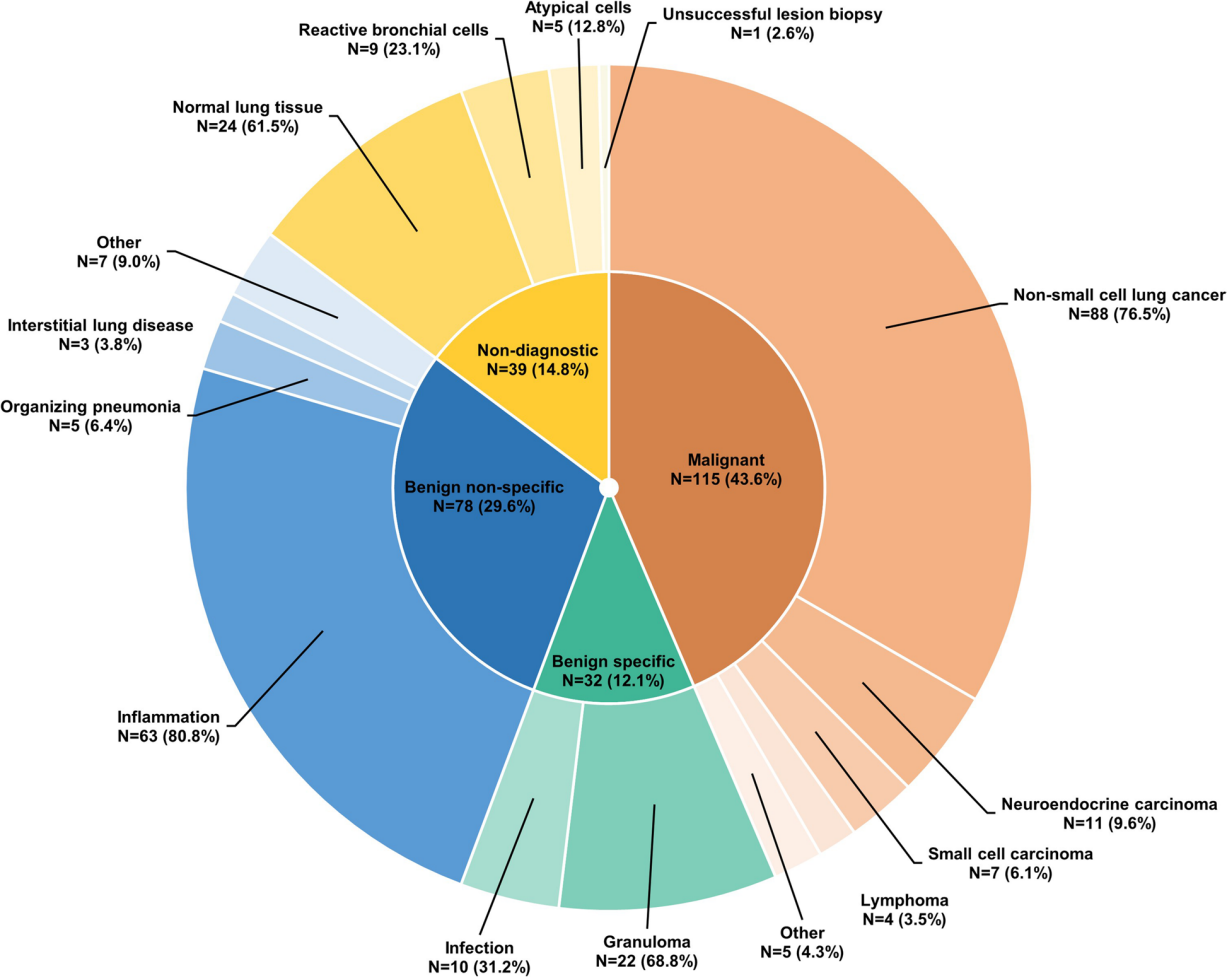
Distribution of index diagnoses (*N* = 264)

Tissue samples were successfully obtained at the index RAB procedure in all but one case (99.6%), which was classified as non-diagnostic. The index RAB procedure led to a malignant diagnosis in 115 patients (43.6%) (Fig. 1). Adenocarcinoma was the most commonly diagnosed malignancy (46.1%).

### Diagnostic yield estimates

The diagnostic yield at the time of the index procedure was 85.2% (95% confidence interval [CI]: [80.9%, 89.5%]) (Table 3) using the first calculation method, whereas the 12-month diagnostic yield was 79.4% (95% CI: [74.4%, 84.3%]). Worst- and best-case bounds on 12-month diagnostic yield were 77.3% (95% CI: [72.2%, 82.3%]) and 79.9% (95% CI: [75.1%, 84.8%]) based on classifying seven inconclusive cases as FN and TN, respectively.

**Table 3:** Diagnostic yield and sensitivity for malignancy

**Diagnostic Outcomes**			***N* = 264**	
			**% (95% CI)**	
Diagnostic yield at index, using first definition			85.2% (80.9%, 89.5%)	
Diagnostic yield at 12 months, using first definition				
Base case			79.4% (74.4%, 84.3%)	
Worst case			77.3% (72.2%, 82.3%)	
Best case			79.9% (75.1%, 84.8%)	
Sensitivity for malignancy at 12 months				
Base case scenario			79.3% (72.7%, 85.9%)	
Worst case scenario			74.2% (67.3%, 81.1%)	
**Comparison of Diagnostic Yield Definitions**				
	**Index Diagnostic yield**		**12-month Diagnostic yield**	
**Index Diagnosis Status**	**First** **Definition**	**Second** **Definition**^**a**^	**First** **Definition**	**Second** **Definition**
Malignant	TP (*N* = 115)	TP (*N* = 115)	TP (*N* = 115)	TP (*N* = 115)
Benign specific	TN (*N* = 32)	TN (*N* = 40)	TN (*N* = 32)	TN (*N* = 32)
Benign non-specific	TN (*N* = 78)	FN (*N* = 70)	TN (*N* = 57), FN (*N* = 14), or inconclusive (*N* = 7)	TN (*N* = 57), FN (*N* = 14), or inconclusive (*N* = 7)
Non-diagnostic	FN (*N* = 39)	FN (*N* = 39)	FN (*N* = 39)	TN (*N* = 22), FN (*N* = 14), or inconclusive (*N* = 3)
**Diagnostic Yield**				
Numerator	225	155	204	226
Denominator	264	264	257	254
Estimate, % (95% CI)	85.2% (80.9%, 89.5%)	58.7% (52.8%, 64.7%)	79.4% (74.4%, 84.3%)	89.0% (85.1%, 92.8%)

CI, confidence interval

^a^Cases of organizing pneumonia and interstitial lung disease were classified as benign specific

Using alternate definitions had a considerable impact on diagnostic yield estimates. The definition from the AQUiRE study, which considered benign non-specific diagnoses as non-diagnostic, resulted in a markedly lower estimate of diagnostic yield at index – 58.7% (95% CI: [52.8%, 64.7%]). The 12-month diagnostic yield calculated using a definition similar to NAVIGATE, which considered follow-up data for cases that were non-diagnostic at index, provided the highest estimate of 89.0% (95% CI: [85.1%, 92.8%]), with worst- and best-case bounds of 85.6% (95% CI: [81.4%, 89.8%]) and 89.4% (95% CI: [85.7%, 93.1%]), respectively. 

### Sensitivity for malignancy

Besides the 115 patients with a malignancy diagnosis at the index RAB procedure, an additional 30 patients had a cancer diagnosis within 12 months (FN). Malignancy could not be ruled out for ten patients (inconclusive cases) – seven due to missing follow-up and three due to an increase in lesion size. Sensitivity for malignancy was 79.3% (95% CI: [72.7%, 85.9%]), with a worst-case scenario of 74.2% (95% CI: [67.3%, 81.1%]). 

Overall prevalence of cancer in the study population was 58.0%, using the 250 patients who had diagnostic follow-up beyond the index procedure as the denominator. 

### Diagnostic yield in subgroups of interest

Diagnostic yield at index was significantly higher when primary lesions were larger (89.8% vs. 81.0% for those with a diameter ≥20 mm and <20 mm, respectively; *p*=0.0455), had a visible bronchus sign (92.3% vs. 81.8%; *p*=0.0296), and were solid or semi-solid (87.3% and 82.6%, respectively, versus 61.5% for pure ground glass lesions; *p*=0.0341) (Table 4). In addition, a significantly higher yield was observed in patients with a pre-procedure probability of malignancy ≥65% (91.8% vs. 79.4%; *p*=0.0052) and for procedures where multiple lesions were biopsied (97.7% vs. 82.8%; *p*=0.0119). Lesion size, pre-procedure probability of malignancy, and biopsy of multiple lesions were also associated with a higher 12-month diagnostic yield.

**Table 4:** Diagnostic yield at index and at 12 months in subgroups of interest

Predictor	Index Diagnostic Yield^a^	*P*-Value	12-month Diagnostic Yield^a^	*P*-Value
**Operator and study site detail**				
Operator specialty				
Interventional pulmonologist	189/226 (83.6%)	0.0742	172/219 (78.5%)	0.4250
Thoracic surgeon	36/38 (94.7%)		32/38 (84.2%)	
≤ 5th case for operator				
No	206/242 (85.1%)	0.8753	187/235 (79.6%)	0.7986
Yes	19/22 (86.4%)		17/22 (77.3%)	
Setting				
Inpatient	16/17 (94.1%)	0.2855	15/17 (88.2%)	0.3503
Outpatient	209/247 (84.6%)		189/240 (78.8%)	
**Patient Characteristics**				
Age ≥ 65				
No	78/86 (90.7%)	0.0817	68/82 (82.9%)	0.3357
Yes	147/178 (82.6%)		136/175 (77.7%)	
Sex				
Female	123/150 (82.0%)	0.0900	116/148 (78.4%)	0.6446
Male	102/114 (89.5%)		88/109 (80.7%)	
Smoking status				
Current smoker	58/72 (80.6%)	0.4870	53/70 (75.7%)	0.6697
Never smoked	58/65 (89.2%)		53/64 (82.8%)	
Prior smoker	107/125 (85.6%)		96/121 (79.3%)	
Unknown/missing	2/2 (100%)		2/2 (100%)	
Emphysema				
No	130/150 (86.7%)	0.4496	119/148 (80.4%)	0.6351
Yes	95/114 (83.3%)		85/109 (78.0%)	
COPD				
No	109/128 (85.2%)	0.9748	101/127 (79.5%)	0.9531
Yes	116/136 (85.3%)		103/130 (79.2%)	
Pre-procedure probability of malignancy^b^ ≥ 65%				
No	104/131 (79.4%)	0.0052	91/127 (71.7%)	0.0023
Yes	112/122 (91.8%)		104/119 (87.4%)	
**Lesion detail**				
Primary lesion solidity				
Pure ground glass	8/13 (61.5%)	0.0341	8/13 (61.5%)	0.1659
Semi-solid	38/46 (82.6%)		33/44 (75.0%)	
Solid	179/205 (87.3%)		163/200 (81.5%)	
Primary lesion location				
Proximal third	22/27 (81.5%)	0.6910	20/27 (74.1%)	0.7278
Middle	71/81 (87.7%)		65/80 (81.3%)	
Peripheral (outer third of the lung)	131/155 (84.5%)		118/149 (79.2%)	
Primary lesion lobe location				
Left lower lobe	29/34 (85.3%)	0.9847	22/32 (68.8%)	0.4541
Left upper lobe	66/79 (83.5%)		59/76 (77.6%)	
Right lower lobe	45/52 (86.5%)		42/51 (82.4%)	
Right middle lobe	15/17 (88.2%)		15/17 (88.2%)	
Right upper lobe	70/82 (85.4%)		66/81 (81.5%)	
Primary lesion bronchus sign				
No	148/181 (81.8%)	0.0296	132/174 (75.9%)	0.0707
Yes	72/78 (92.3%)		67/78 (85.9%)	
Distance from lesion to pleura, mm				
0	48/57 (84.2%)	0.9298	45/56 (80.4%)	0.7559
< 10	31/36 (86.1%)		28/34 (82.4%)	
≥ 10	101/117 (86.3%)		87/113 (77.0%)	
Primary lesion size^c^ < 20 mm				
No	114/127 (89.8%)	0.0455	107/125 (85.6%)	0.0164
Yes	111/137 (81.0%)		97/132 (73.5%)	
**Procedural details**				
Biopsy of multiple lesions				
No	183/221 (82.8%)	0.0119	164/214 (76.6%)	0.0154
Yes	42/43 (97.7%)		40/43 (93.0%)	
R-EBUS image type				
Concentric	71/79 (89.9%)	0.2455	67/79 (84.8%)	0.2417
Eccentric	72/86 (83.7%)		66/85 (77.6%)	
ROSE used				
No	82/92 (89.1%)	0.1912	72/91 (79.1%)	0.9400
Yes	143/172 (83.1%)		132/166 (79.5%)	
Monarch Platform software version				
2.1.3	49/55 (89.1%)	0.8132	43/54 (79.6%)	0.9816
2.1.4	45/53 (84.9%)		40/51 (78.4%)	
2.1.5	62/73 (84.9%)		54/69 (78.3%)	
2.1.6	69/83 (83.1%)		67/83 (80.7%)	
Biopsy forceps used				
No	62/77 (80.5%)	0.1666	57/75 (76.0%)	0.3903
Yes	163/187 (87.2%)		147/182 (80.8%)	
Cytology brush used				
No	184/219 (84.0%)	0.2220	165/212 (77.8%)	0.1833
Yes	41/45 (91.1%)		39/45 (86.7%)	
Aspiration needle used				
No	7/9 (77.8%)	0.5216	6/9 (66.7%)	0.3373
Yes	218/255 (85.5%)		198/248 (79.8%)	

*COPD* Chronic obstructive pulmonary disease, *R-EBUS* Radial endobronchial ultrasound, *ROSE* Rapid onsite evaluation

^a^Calculated using the first definition

^b^Calculated using an adapted Mayo Clinic model

^c^Size was calculated as the mean from long and short axes dimensions when both were reported and as the long axis dimension otherwise

### Complications

Twenty patients (7.6%) had at least one device or procedure-related complication. Pneumothorax was the most commonly recorded complication (*n*=15, 5.7%). Ten of these cases (3.8%) required the placement of a chest tube, of which two also had respiratory failure reported. Four patients (1.5%) experienced bleeding events requiring intervention, and three had other device or procedure-related complications. There were no device or procedure-related deaths.

Pneumothorax rates were significantly higher when the RAB was performed by a thoracic surgeon (15.8% vs. 4.0% for interventional pulmonologists; *p*=0.0113), when primary lesions did not have a bronchus sign (8.3% vs. 0.0%; *p*=0.0067) or were located either in the inner or outer third of the lung (7.4% and 8.4%, respectively vs. 0.0% for middle third; *p*=0.0085), and when rapid onsite evaluation (ROSE) was not used (9.8% vs. 3.5%; *p*=0.0492) (Table 5).

**Table 5:** Pneumothorax rate in subgroups of interest

Predictor	Pneumothorax Rate	*P*-Value
**Operator and study site detail**		
Operator specialty		
Interventional pulmonologist	9/226 (4.0%)	0.0113
Thoracic surgeon	6/38 (15.8%)	
≤ 5th case for operator		
No	13/242 (5.4%)	0.3609
Yes	2/22 (9.1%)	
Setting		
Inpatient	2/17 (11.8%)	0.2499
Outpatient	13/247 (5.3%)	
**Patient Characteristics**		
Age ≥ 65		
No	3/86 (3.5%)	0.3986
Yes	12/178 (6.7%)	
Sex		
Female	10/150 (6.7%)	0.5932
Male	5/114 (4.4%)	
Smoking status		
Current smoker	5/72 (6.9%)	0.8992
Never smoked	3/65 (4.6%)	
Prior smoker	7/125 (5.6%)	
Unknown/missing	0/2 (0%)	
Emphysema		
No	8/150 (5.3%)	0.7943
Yes	7/114 (6.1%)	
COPD		
No	7/128 (5.5%)	1.0000
Yes	8/136 (5.9%)	
Pre-procedure probability of malignancy^a^ ≥ 65%		
No	5/131 (3.8%)	0.2749
Yes	9/122 (7.4%)	
**Lesion detail**		
Primary lesion solidity		
Pure ground glass	1/13 (7.7%)	0.1570
Semi-solid	5/46 (10.9%)	
Solid	9/205 (4.4%)	
Primary lesion location		
Proximal third	2/27 (7.4%)	0.0085
Middle	0/81 (0%)	
Peripheral (outer third of the lung)	13/155 (8.4%)	
Primary lesion lobe location		
Left lower lobe	1/34 (2.9%)	0.7230
Left upper lobe	6/79 (7.6%)	
Right lower lobe	4/52 (7.7%)	
Right middle lobe	1/17 (5.9%)	
Right upper lobe	3/82 (3.7%)	
Primary lesion bronchus sign		
No	15/181 (8.3%)	0.0067
Yes	0/78 (0%)	
Distance from lesion to pleura, mm		
0	6/57 (10.5%)	0.5044
< 10	2/36 (5.6%)	
≥ 10	7/117 (6.0%)	
Primary lesion size^b^ < 20 mm		
No	6/127 (4.7%)	0.6006
Yes	9/137 (6.6%)	
**Procedural details**		
Biopsy of multiple lesions		
No	13/221 (5.9%)	1.0000
Yes	2/43 (4.7%)	
R-EBUS image type		
Concentric	5/79 (6.3%)	1.0000
Eccentric	5/86 (5.8%)	
ROSE used		
No	9/92 (9.8%)	0.0492
Yes	6/172 (3.5%)	
Monarch Platform software version		
2.1.3	7/55 (12.7%)	0.0891
2.1.4	3/53 (5.7%)	
2.1.5	3/73 (4.1%)	
2.1.6	2/83 (2.4%)	
Biopsy forceps used		
No	5/77 (6.5%)	0.7716
Yes	10/187 (5.3%)	
Cytology brush used		
No	15/219 (6.8%)	0.0820
Yes	0/45 (0%)	
Aspiration needle used		
No	1/9 (11.1%)	0.4143
Yes	14/255 (5.5%)	

*COPD* Chronic obstructive pulmonary disease, *R-EBUS* Radial endobronchial ultrasound, *ROSE* Rapid onsite evaluation

^a^Calculated using an adapted Mayo Clinic model

^b^Size was calculated as the mean of the long and short axes dimensions when both were reported and as the long axis dimension otherwise

## Discussion

The present retrospective multicenter study is the largest to date evaluating the diagnostic yield in patients undergoing RAB for the diagnosis of pulmonary lesions. Moreover, the results herein reported represent the first data on the Monarch Platform obtained exclusively in a community setting. 

Use of the Monarch RAB Platform led to high diagnostic yield, despite a high proportion of traditionally challenging lesions which have historically been associated with lower diagnostic accuracy of bronchoscopy [3, 16, 19]. Primary lesions with a mean diameter <20 mm (51.9%), absent bronchus sign (69.9%), located in the upper lobe (61.0%) and in the peripheral third of the lung (58.9%) were in the majority. Moreover, an appreciable proportion of primary lesions were sub-solid in nature (22.4%). 

Our study is the first to evaluate diagnostic yield using several different approaches that have been commonly reported in the literature. This was intended to provide transparency in outcome assessment and help overcome the challenge of performing valid comparisons across bronchoscopy studies which to date have used non-standardized definitions. Two prior studies have explored the 12-month diagnostic yield of the Monarch Platform, with resulting rates of 74.1% and 77.0%, respectively [9, 10]. Using a similar approach, we found a comparable diagnostic yield after 12 months of follow-up (79.4%). 

Similar results have been reported for another RAB platform that uses shape-sensing technology. In a single-site study of 29 patients, twenty-three samples were diagnostic (malignant or benign diagnoses), yielding a diagnostic yield of 79.3% at index [13]. Data from 69 patients enrolled in a recent multicenter prospective study showed a diagnostic yield of 83% [15]. We obtained an index diagnostic yield of 85.2% using a similar methodological approach. In addition, our primary 12-month diagnostic yield estimate (79.4%) was comparable to that reported for the shape-sensing RAB platform at 12 months (81.7%) [14]. 

Our findings suggest that RAB may result in a higher diagnostic yield when compared with traditional EMN bronchoscopy. NAVIGATE, the largest study to evaluate the diagnostic yield of EMN bronchoscopy for pulmonary lesions using the superDimension navigation system (Medtronic, Minneapolis, Minnesota), demonstrated a rate of 72.9% at 12 months [17]. To estimate the diagnostic yield, all cases negative for malignancy at index were followed through 12 months to determine the true diagnosis. We found a higher diagnostic yield (89.0%) when attempting to replicate NAVIGATE’s approach. Our higher rate could be partially due to differences in patient and lesion characteristics, and well as in the use of concurrent imaging across the two studies. In addition, unlike the NAVIGATE study, we did not consider follow-up data for patients with benign specific diagnoses at index – they were always considered TN. The impact of this difference in methodology on our estimate was minimal (diagnostic yield is 87.6% when considering follow-up data for these patients). The difference in diagnostic yield between our study and NAVIGATE assumes particular significance when considering that our study was based on initial cases of RAB use (four of the six operators were new users of the platform and no run-in period was considered) while NAVIGATE was conducted after the superDimension technology had relatively matured. 

Our diagnostic yield also compares favorably to that reported in the AQUiRE study, which used a variety of guidance technologies (rEBUS, EMN, virtual bronchoscopy, etc.) and sampling tools (brush, needle, forceps, lavage, etc.) [16]. This study used the most stringent method to calculate diagnostic yield (whereby only malignant or benign specific diagnoses were considered diagnostic), yielding an estimate of 53.7% based on 581 peripheral lesions. We found a higher diagnostic yield (58.7%) with the Monarch Platform and a similar calculation method.

By limiting the variability in diagnostic yield estimates arising from the use of different calculation methods, we expect to have improved the validity of comparisons made between our study and select published bronchoscopy studies using similar definitions. Nonetheless, heterogeneity across studies, namely in terms of patient and lesion characteristics, remains a significant influence on estimates of diagnostic yield and needs to be further contextualized. Notably, the proportion of lesions in our study with a visible bronchus sign (30.1% for primary lesions) was appreciably lower than in previous bronchoscopy studies (48.5% to 75.0%) [9, 10, 13, 17]. The presence of a bronchus sign has been shown to significantly increase the diagnostic yield of bronchoscopic procedures [20], as was observed in our study (Table 4). Furthermore, the prevalence of cancer in our community setting (58.0%) was also lower than reported in prior research. At 12 months, the NAVIGATE study found a 67% prevalence of malignancy [17], while studies on shape-sensing RAB reported rates of 65.4% and 73.9% [14, 15]. Since an increased cancer prevalence is expected to improve the diagnostic yield of the bronchoscopic procedure [11], higher diagnostic yields may be attainable via RAB in populations with a higher pre-procedure probability of malignancy. Operator feedback from our study has also suggested that the shift in patient selection to include more cases with lower pre-procedure probability of malignancy or traditionally challenging lesion anatomy is at least partly due to gaining experience with the latest advances in RAB technology, which has provided increased confidence to achieve a successful biopsy in these more difficult cases. 

The size of pulmonary nodules has been consistently identified as a predictor of diagnostic yield [10, 14, 17, 21]. Consistent with previous studies, we found that the diagnostic yield was significantly improved for larger lesions, both at index (89.8% and 81.0% when primary lesions had a mean diameter ≥20 mm and <20 mm, respectively) and after 12 months of follow-up (85.6% vs. 73.5%, respectively). Nonetheless, the diagnostic yield estimates for smaller lesions are noteworthy, as they represent a considerable improvement over prior reports of guided bronchoscopic biopsy (60.9%) [3]. Besides greater accuracy with smaller lesions, our study also showcases the utility of RAB to sample and diagnose other traditionally challenging lesions. High diagnostic yields at index (>80%) were obtained even in lesions in the outer one third of the lung, right upper lobe and with no visible bronchus sign. Conversely, the diagnostic yield at index for pure ground glass lesions was lower (61.5%), with higher yields being obtained for solid (87.3%) and semi-solid (82.6%) lesions. 

Consistent with previous literature [11, 12], our findings show that diagnostic yield estimates vary considerably depending on the calculation approach adopted, highlighting the need for a standard method of computing this metric. At index, the use of two definitions resulted in an absolute difference of 26.5% between the first and second estimates (85.2% and 58.7%, respectively). As expected, the methodology used in NAVIGATE resulted in the highest estimate because patients with non-diagnostic bronchoscopic pathology at index could be classified as TN if they did not have a malignant diagnosis during follow-up. Accordingly, prior analyses have noted that this methodology is relatively insensitive to population level variation of cancer prevalence and provides the highest diagnostic yield estimates [11]. 

Pneumothorax was the most common complication arising from the RAB procedure in our study; we observed a higher rate in our real-world setting (5.7%) compared to previous studies involving the Monarch platform (1.6% to 3.7%) [8–10]. Notably, all 15 pneumothorax events in our study occurred when the primary lesion had no visible bronchus sign on pre-procedure CT scans. The lack of a bronchus leading directly to the lung lesion (i.e., bronchus sign) may increase the complexity of navigating to the lesion and collecting a tissue sample. Therefore, our higher pneumothorax rate may be partially explained by the markedly lower proportion of lesions with a bronchus sign, as compared to previous studies [8–10]. In addition, the inclusion of a thoracic surgeon as one of the study operators may have contributed to our higher pneumothorax rate. In fact, a rate closer to those historically reported for Monarch was found in the subset of procedures performed by interventional pulmonologists (4.0%), while the rate was 15.8% for procedures performed by a thoracic surgeon. Procedures performed by the surgeon were characterized by a significantly lower use of ROSE (21.1% vs. 72.6%) (Additional file Table 3) and higher diagnostic yields at index and 12 months. We also found a numerically higher rate of pneumothorax in sub-solid lesions, which accounted for approximately 22% of the primary lesions biopsied in our study. These findings may point to the use of a more aggressive biopsy approach with advancing RAB technology that may explain the higher pneumothorax rate in our study. 

The major limitation of this study is the retrospective nature of data collection. However, the use of a pre-specified protocol with clear algorithms for classification of pathology at index and calculations of diagnostic yield is expected to have decreased subjectivity, improving the robustness of the study results. In addition, while we aimed to replicate the key features of diagnostic yield definitions used in published bronchoscopy literature, minor differences in interpretation may have occurred, affecting comparisons across studies. These could potentially include differences in how specific pathology results were classified at index or the criteria for patients with a non-malignant index diagnosis to be followed up for diagnostic yield assessment. Head-to-head studies of bronchoscopic technologies are needed to more accurately compare diagnostic yields of emerging technologies.

## Conclusion

RAB demonstrated a high diagnostic yield in the largest study to date, despite representing a real-world community population with a relatively low pre-procedure probability of malignancy and where traditionally challenging lesions, such as those < 20 mm, peripherally located, and without a bronchus sign on CT scan, were in the majority.

## Supplementary Information

**Additional file 1: Table 1. **Classification of index pathology results as malignant, benign specific, benign non-specific, and non-diagnostic.

**Additional file 2: Table 2. **Secondary Lesion Characteristics.

**Additional file 3: Table 3. **Patient, Lesion, and Procedural Detail by Operators’ Medical Specialty.

## Data Availability

The datasets used and/or analyzed during the current study are available from the corresponding author on reasonable request.

## Abbreviations

CI: Confidence interval
CT: Computed tomography
EMN: Electromagnetic navigation
FN: False negative
RAB: Robot-assisted bronchoscopy
R-EBUS: Radial endobronchial ultrasound
ROSE: Rapid onsite evaluation
TN: True negative
TP: True positive
US: United States
