# Supplementary material for: Diagnostic outcomes of robotic-assisted bronchoscopy for pulmonary lesions in a real-world multicenter community setting
Source: BMC Pulm Med. 2023 May 9;23:161. doi: 10.1186/s12890-023-02465-w (PMC10170714; doi:10.1186/s12890-023-02465-w)
Supplement: Supplementary file 1 — Additional file 1: Table 1. Classification of index pathology results as malignant, benign specific, benign non-specific, and non-diagnostic. [file 12890_2023_2465_MOESM1_ESM.pdf]

Additional File Table 1 – Classification of index pathology results as malignant, benign specific, benign non-specific, and non-diagnostic

Positive for Malignancy at Index		Negative for Malignancy at Index	
**Malignant**	**Benign Specific**	**Benign Non-Specific**	**Non-Diagnostic**
Non-small cell lung cancer	Infection / abscess	Inflammation	Normal lung tissue
Adenocarcinoma	Bacterial	Acute/benign inflammation	Bronchial epithelium
Adenocarcinoma in situ	Fungal	Chronic inflammation	Hemorrhage
Squamous cell carcinoma	Viral	Inflammatory tissue	Macrophages
Large cell carcinoma	Granuloma	Neutrophilic inflammation	Blood clots
Large cell neuroendocrine carcinoma	Granulomatous inflammation	Lymphocytes	Focally anthracotic alveolated pulmonary parenchyma
Adenosquamous carcinoma	Necrotizing (caseating)	Organizing pneumonia	Benign tissue
Sarcomatoid carcinoma	Non-necrotizing	Multinucleated giant cell(s)	Reactive bronchial cells
Small cell carcinoma	Hamartoma	Squamous dysplasia/ metaplasia	Atypical cells[a]
Small cell carcinoma (oat cell cancer)	Benign metastasizing leiomyoma	Radiotherapy changes	
Combined small cell carcinoma		Asbestos fibrosis	
Neuroendocrine carcinoma		Iron pill aspiration	
Other types of lung tumors		Fibroelastic scar	
Mesothelioma		Interstitial lung disease	
Adenoid cystic carcinoma of the lung		Bronchogenic cyst	
Lung sarcomas			
Lymphomas			
Germ cell tumors			
Teratomas			
Thymomas and thymic carcinoma			
Other malignant cells (unable to characterize)			
Metastatic carcinoma			
From a hematological origin			
Solid tumor origin			
Other - deemed malignant based on multidisciplinary review			

[a]Insufficient evidence for malignant diagnosis
