# Supplementary material for: Diagnostic outcomes of robotic-assisted bronchoscopy for pulmonary lesions in a real-world multicenter community setting
Source: BMC Pulm Med. 2023 May 9;23:161. doi: 10.1186/s12890-023-02465-w (PMC10170714; doi:10.1186/s12890-023-02465-w)
Supplement: Supplementary file 2 — Additional file 2: Table 2. Secondary Lesion Characteristics. [file 12890_2023_2465_MOESM2_ESM.pdf]

Additional File Table 2 – Secondary Lesion Characteristics

Lesion Characteristic	Total (N=48)
Lesion size[a], mm	15.5 (5.5–53.5)[b]
Lesion size[a] <20 mm	30/47 (63.8%)
Location	
Proximal third	9/47 (19.2%)
Middle	14/47 (29.8%)
Peripheral (outer third of the lung)	24/47 (51.1%)
Distance from closest edge to pleura, mm	18.5 (0.0–91.0)[c]
Lesion lobe location	
Right upper lobe	15/47 (31.9%)
Right middle lobe	5/47 (10.6%)
Right lower lobe	12/47 (25.5%)
Left upper lobe	10/47 (21.3%)
Left lower lobe	5/47 (10.6%)
Visible bronchus leading to lung lesion	20/47 (42.6%)
Nodule type	
Solid	25/47 (53.2%)
Subsolid	22/47 (46.8%)
Pure ground glass (non-solid)	6 (27.3%)
Semi-solid	16 (72.7%)
Margin specifications	
Spiculated	17/42 (40.5%)
Smooth	9/42 (21.4%)
Lobulated	16/42 (38.1%)
Calcification	0/47 (0%)

Results are displayed n (%) or median (minimum–maximum)

[a]Size was calculated as the mean of the long and short axes dimensions when both were reported and as the long axis dimension otherwise

[b]Based on 47 non-missing values

[c]Based on 44 non-missing values
