# Supplementary material for: Diagnostic outcomes of robotic-assisted bronchoscopy for pulmonary lesions in a real-world multicenter community setting
Source: BMC Pulm Med. 2023 May 9;23:161. doi: 10.1186/s12890-023-02465-w (PMC10170714; doi:10.1186/s12890-023-02465-w)
Supplement: Supplementary file 3 — Additional file 3: Table 3. Patient, Lesion, and Procedural Detail by Operators’ Medical Specialty. [file 12890_2023_2465_MOESM3_ESM.pdf]

Additional File Table 3 – Patient, Lesion, and Procedural Detail by Operators' Medical Specialty

	Interventional Pulmonology (N=226)	Thoracic Surgery (N=38)	*P*-Value
**Study site detail**			
Setting			
Inpatient	15 (6.6%)	2 (5.3%)	1.0000
Outpatient	211 (93.4)	36 (94.7)	
**Patient Characteristics**			
Emphysema			
No	122 (54.0%)	28 (73.7%)	0.0327
Yes	104 (46.0%)	10 (26.3%)	
COPD			
No	108 (47.8%)	20 (52.6%)	0.6030
Yes	118 (52.2%)	18 (47.4%)	
Prior lung surgery			
No	205 (90.7%)	30 (78.9%)	0.0463
Yes	21 (9.3%)	8 (21.1%)	
**Lesion detail**			
Primary lesion solidity			
Subsolid	43 (19.0%)	16 (42.1%)	0.0029
Solid	183 (81.0%)	22 (57.9%)	
Primary lesion location			
Proximal third	17 (7.6%)	10 (26.3%)	0.0050
Middle	72 (32.0%)	9 (23.7%)	
Peripheral (outer third of the lung)	136 (60.4%)	19 (50.0%)	
Primary lesion bronchus sign			
No	159 (71.9%)	22 (57.9%)	0.0878
Yes	62 (28.1%)	16 (42.1%)	

Lesions at the pleura			
No	123 (71.5%)	30 (78.9%)	0.4234
Yes	49 (28.5%)	8 (21.1%)	
Primary lesion size[a] <20 mm			
No	105 (46.5%)	22 (57.9%)	0.2211
Yes	121 (53.5%)	16 (42.1%)	
**Procedural details**			
ROSE used			
No	62 (27.4%)	30 (78.9%)	<0.0001
Yes	164 (72.6%)	8 (21.1%)	
Monarch Platform software version			
≤2.1.4	81 (35.8%)	27 (71.1%)	<0.0001
>2.1.4	145 (64.2%)	11 (28.9%)	

COPD, chronic obstructive pulmonary disease; ROSE, rapid onsite evaluation

[a]Size was calculated as the mean of the long and short axes dimensions when both were reported and as the long axis dimension otherwise
